# A Model of Trust within the Mother-Midwife Relationship: A Grounded Theory Approach

**DOI:** 10.1155/2020/9185313

**Published:** 2020-10-15

**Authors:** Firoozeh Mirzaee, Mahlagha Dehghan

**Affiliations:** ^1^Nursing Research Center, Razi School of Nursing and Midwifery, Kerman University of Medical Science, Kerman, Iran; ^2^Nursing Research Center, Department of Critical Care Nursing, Razi Faculty of Nursing and Midwifery, Kerman University of Medical Sciences, Kerman, Iran

## Abstract

**Introduction:**

The mother-midwife relationship is a good experience during childbirth, but there is a lack of evidence about the trustful relationship between mothers and healthcare providers during labor and birth in Iran. The current study aimed to discover how a trustful mother-midwife relationship is formed during a vaginal delivery.

**Methods:**

Twenty-nine women who had a vaginal delivery, midwives, and obstetricians participated in this qualitative research with the grounded theory method. Data were collected using semistructured interviews and observations. Open, axial, and selective coding was used for data analysis. *Findings*. The main category of “seeking trust in midwife” and three subcategories of “effective interaction,” “attempt to access to healthcare provider”, and “playing an active role in birth” were extracted from the data.

**Conclusion:**

According to the findings, mothers tried to gain action/interaction strategies and increase healthcare providers' trusts during vaginal delivery. It is essential to consider the factors that improve or disrupt this relationship.

## 1. Introduction

Trust is vital in a mother-midwife relationship, without which it is impossible to meet the requests of mothers efficiently and to improve midwifery care [1–3]. A trusting relationship includes opportunities for personal growth and improvement [4], self-assurance, communication, honor, mutual respect, skill, justice, and privacy. Trust is also multidimensional and active [5]. Therefore, the cooperative relationships among the midwife, the mother, and the medical system permit a safe and active midwifery practice [6].

As there is regularly a gap between the mothers' prospects and experiences of childbirth, the midwife must provide mothers suitable information on labor development, support her to participate in the childbirth experience actively, and continue the process of delivery for a positive birth experience [7]. Mothers should trust the professionals who care for them and further be involved in decision making [4, 8, 9]. All these trustful relationships may result in a positive experience of childbirth, development of mental health, and improvement of the quality of life of mothers [10].

Different studies mentioned specifically the quality of mother-midwife relationship. Lewis et al. reported that the concept of trust within the mother-midwife relationship was first developed by midwife competency, the kind of communication, the continuity of care, and the mother's ability to make decisions [9]. Boyle et al. confirmed that a mother-midwife relationship mostly focused on biomedical aspects of the birth and midwives had less attention to psychosocial and emotional needs of mothers during pregnancy and childbirth [8]. In another study, midwives reported responsibility, presence, and a calm mutual relationship as some aspects of midwifery care during birth [11]. Bradfield et al. showed that adaptability and self-awareness improved the ability of midwife not to leave women alone, and midwives emphasized trusting relationship with women during childbirth [12]. Woman-centered care in pregnancy and childbirth emphasized that there should be a calm, trustful, and safe atmosphere during the birthing process [13].

Therefore, while a trustful communication during childbirth is a component of best practice [14], some midwives do not care about it [8, 15]. The literature review of quantitative and qualitative research discloses overall information about trust in maternity care, but there is a lack of evidence about the trust between mothers and healthcare providers during labor and birth in Iran. As it is obvious, a combination of manmade, contextual, and political factors influences the formation of the trust within the mother-midwife relationship. However, the trust in the mother-midwife relationship should be based on the best available evidence [16]. Therefore, this study aimed to discover how a trustful mother-midwife relationship is formed in Iran.

## 2. Methods

### 2.1. Study Design

This qualitative research was done with a grounded theory approach, which looks at qualitative data systematically and aims to generate theory. Categories, codes, and coding are important aspects of the grounded theory approach. The research principle behind the grounded theory method combines both induction and deduction [17]. Therefore, this approach permitted the researchers to explore the patterns of behavior among participants and to describe the social processes shaping participants' interactions.

### 2.2. Setting and Participants

There is no specific rule in qualitative research to estimate the number of participants and sampling continued until data saturation with no new information [18]. In the present study, the saturation was achieved after interviewing 12 mothers with vaginal deliveries, 11 midwives, and six obstetricians working at private or public hospitals in Kerman, Iran. We started purposive sampling by interviewing mothers, midwives, and obstetricians (theoretical sampling). The inclusion criteria were speaking in Farsi, vaginal delivery, working at public or private hospitals, and healthy newborn. We selected participants with different parity, ages, educational levels, and socioeconomic status for maximum variation. Fourteen women and 12 midwives took part in the study; two women refused to participate in the survey, and one midwife withdrew from the study. All interviews were conducted in the postpartum ward in hospitals except for one, which was done at home. The sampling and analysis lasted from March 2016 to April 2017.

### 2.3. Data Collection

Before performing the interview, the researcher (FM), the interviewer, and an experienced midwife explained the study objectives. The semistructured in-depth interview was used to collect the data. The interviews began with open-ended questions: “how did you/women experience trust in vaginal delivery? What was the meaning of trust”? Then, the interviewer applied additional questions such as “Would you please tell more about this experience”? All of the interviews were voice recorded using an MP3 recorder (model ICD-PX470) and then were transcribed verbatim. The interviews were recorded and analyzed in the Persian language, and then, the final report was translated into English. Data collection was continued until data saturation. The last four interviews did not add any extra information. Each interview lasted between 45 and 60 minutes. Written narratives were also used, so a notebook was delivered to each of the participants (if they were willing), and they were requested to write their experiences and return the notebook to the researcher. The researcher also wrote field notes and memos throughout and after the interviews, which were used as complementary data.

### 2.4. Data Analysis

**Figure 1:**
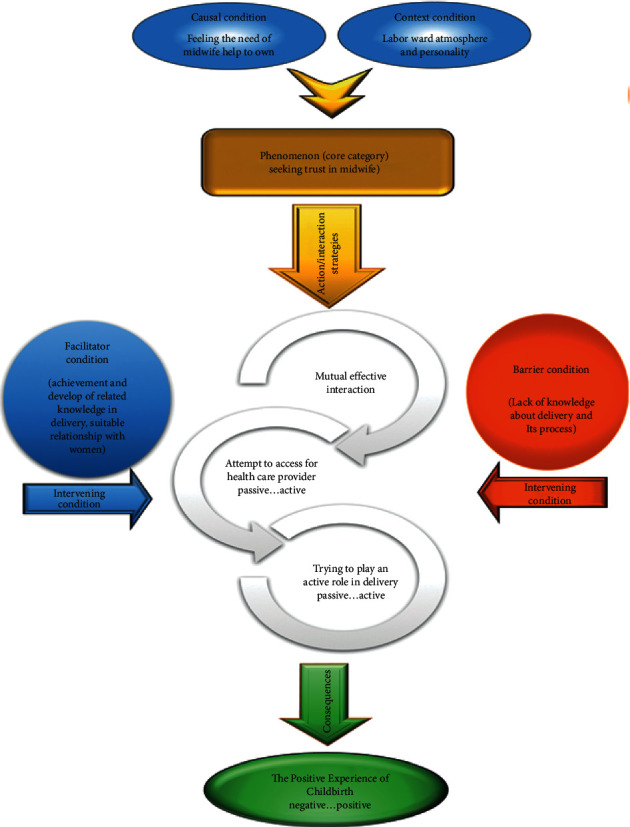
Theoretical scheme of trust in childbirth.

The data analysis was based on the following phases recognized by Strauss and Corbin: (1) open coding, (2) axial coding, (3) selective coding, and (4) development of the theory [17]. Concurrent data collection and analysis were used. Data analysis included the processes of coding and conceptualizing data, with a constant comparison between data and memos. In addition, theoretical sensitivity and constant comparison among codes, categories, memos, and literature were maintained throughout all the stages. Inductive and deductive thinking was based on Strauss and Corbin's coding paradigm [17]: permissible identification of the main perception of trust in childbirth with its axially coded subindicators. During data collection and analysis, the researcher continued openly the stream of ideas, expectations, and experiences revealed by the participants. When reading the data, we tried to limit opinions defined through the lens of the participant. We used triangulation of sources because women and midwives both took part in the study. The initial concepts allowed for the process of data collection, coding, and target analysis. Following the constant comparative analysis method, the participants' experiences were systematically compared and contrasted with those of the initial cases. The iteration between data and concepts ended when enough categories and associated concepts had been defined to explain the factors affecting core category. Theoretical saturation was achieved when no additional data were collected and added to the set of concepts and categories. The theoretical model is empirically valid because it can consider the unique data of each case in the study and generalize patterns across the cases. The study participants corrected and clarified drafts of findings and the theoretical model. To this end, each interview was immediately transcribed verbatim. Nonverbal gestures such as crying, smiling, and silence were also noticed. Then, transcripts were transferred to MAXQDA software (version 2007) to manage the data. Open, axial, and selective coding and constant comparative analysis were applied to the data. First, we listened to each interview carefully, read the transcript of every interview word by word to capture key thoughts and concepts, and coded them openly. In this stage, the codes, the categories, and memos related to the abstract and theoretical ideas emerged from the data. Data were compared with axial coding to make relations between categories and their subcategories. Three categories were formed, and a paradigm model was developed for categories. Components of the paradigm model were (a) phenomenon, (b) contextual, causal, and intervening conditions, (c) action/interaction strategies for managing phenomenon, and (d) consequences. Lastly, the main category was identified in the selective coding stage (Figure 1). The first researcher (FM) did the three stages of coding, and the other researcher (MD) repeated the analysis and contradictions were resolved by consensus.

### 2.5. Data Trustworthiness

Trustworthiness, a critical aspect of qualitative research, includes how to judge the similarities and differences among categories. The trustworthiness of the data was tested using Lincoln and Guba's criteria [19]. Reflective field notes of the research team strengthened the trustworthiness of the data. Credibility was achieved through member checking and peer checking. The participants received a full transcript of their coded interviews to decide whether the codes and categories were in agreement with their experiences. Then, one external expert performed the peer check. Maximum variation in sampling (considering age, parity, years of work experience in a hospital, and their workplace) also improved the confirmability and credibility of the data. Peer check methods achieved dependability, and the first researcher conducted all the interviews. Transferability of the findings was gained as the results were apparent and logical for three women and two midwives not participated in the study.

### 2.6. Ethical Consideration

The Ethics Committee of the Kerman University of Medical Science approved this study (ethical code: IR.KMU.REC.1396.1701). Written informed consent was received from all participants before the interviews. The researchers have protected anonymity and confidentiality by assigning numbers to participants and eliminating all possible recognizable data during the transcription of interviews. Similarly, password-protected folders containing anonymized data were available only to the research team. The study purpose was explained, and all participants permitted the author for the audio recording of the interviews. The right to withdraw from the study at any time and ethical commitments were described in all interviews.

## 3. Results

### 3.1. Sample Characteristics

The age groups of mothers, midwives, and obstetricians were 18–43, 22–53, and 35–52 years, respectively. The work experiences of the midwives and obstetricians also ranged from 1–34 and 1–18 years, respectively (Table 1).

One main category and three subcategories emerged from the data. The main category was “seeking trust in midwife.” The causal, intervening, and contextual conditions shaped this phenomenon. The action/interaction strategies used by mothers resulted in “formation of childbirth experience” as a consequence. From now on, quotes are shown in italics and subjects are identified with Mo, M, and O for mothers, midwives, and obstetricians, respectively.

In this study, we achieved a dynamic model for trusting healthcare providers, especially midwives. This model may enable mothers and midwives to form a trusting relationship. “Seeking trust in midwife,” “effective mutual interaction,” “an attempt to access to healthcare providers,” and “playing an active role in birth” are essential components of this model.

### 3.2. Core Category: Seeking Trust in Midwife

“Seeking trust in midwife” was the main category that emerged from the participants' experiences. They behave complexly to achieve this ideal. They manage effective interaction with a healthcare provider, then try to access the healthcare provider, and try to play an active role in birth. The most influential approach expressed by participants was the trust between women and midwives.

“I trusted my midwife. When I arrived at the ward, interacted with a midwife, asked her some questions, I did whatever she asked me to make the childbirth easier” (Mo5, 28 years old, first childbirth).

*“Trust in doctors and midwives is the cornerstone in vaginal birth and participation in childbirth decision-making is important for women” (M8*, *39 years old, and ten years of work experiences)*.

According to the paradigm model, the category of “the need for taking part in childbirth” was the causal condition for this phenomenon that occurs in the context of labor ward and personality characteristics of mothers and midwives. Participants applied several strategies to organize this phenomenon. This category was supported by three subcategories, including “effective mutual interaction,” “an attempt to access healthcare providers,” and “playing an active role in childbirth.” According to the participants' experiences, these subcategories were the necessary steps in the continuity of trust between mothers and midwives. Eventually, “formation of childbirth experience” was the consequence of the action/interaction strategies.

### 3.3. Causal Conditions

Participants felt that midwives should help them in their childbirths, so they decided to trust them. Participants also desired to take part in their childbirths *“I should deliver a healthy baby, and then I should help my midwife in the birth process” (Mo4, 32 years old, the second birth).*

*“Mothers feel that they should take part in their childbirths” (O4*, *41 years old, six years of work experiences)*.

### 3.4. Contextual Conditions

The participants frequently talked about contextual conditions affecting the decisions made. According to participants' experiences, the contextual conditions were “the labor ward atmosphere and personality characteristics of mothers and midwives.” Labor ward atmosphere such as the friendly relationship between mothers and healthcare providers, crowdedness of the ward, the small number of midwives, and their characteristics such as being introverted or extroverted would affect the decisions about birth.

“The midwife didn't talk to me and had no relationship with me. She was an introverted person; even she didn't tell me about what I had to do during my childbirth process.” (Mo4, 32 years old, the second birth).

*“Sometimes, the ward is too crowded for us to talk with or to help mothers. Therefore, we can't guide them” (M6*, *41 years old, 12 years of work experiences)*.

### 3.5. Action/Interaction Strategies

Action/interaction strategies are purposeful acts toward a problem to affect the process of trust between mothers and midwives. There were three main subcategories related to action/interaction strategies, including “effective mutual interaction,” “an attempt to access healthcare providers,” and “playing an active role in birth.”

#### 3.5.1. Mutual Effective Interaction

First, mothers tried to interact with healthcare providers. The communication between midwives and mothers is a key aspect of care. In addition, the use of body language manages the condition through appropriate dialogues between midwives and mothers and makes a meaningful communication.

“*I tried to talk to personnel and communicate with them so that they could help me later because no one will help us without communication” (Mo9, 38 years old, third birth).*

Some participants respected the midwives and obstetricians and needed to be respected by them.

*“As a mother respects us, we must respect her, because the first step to help her childbirth is mutual respect and good relationship” (O5, 52 years old, with 13 years of work experiences)*.

#### 3.5.2. An Attempt to Access a Healthcare Provider

Mothers tried to access healthcare providers for the childbirth. Participants frequently sought for continual monitoring and tried to keep the healthcare providers close to themselves.

“I liked to be under close supervision and control of the healthcare providers. I was worried about problems that might have occurred for my fetus and mine. I tried to seek one who could take care of my fetus and mine” (Mo10, 25 years old, first birth).

The mothers also tried to attract the healthcare providers, which makes them relaxed and secure.

*“We must pay attention to mothers during childbirth because it shows that we are taking care of them and their fetus. It provides comfort for them, and they feel that somebody is beside them, and they are not alone” (M10, 50 years old, with 33 years of work experiences)*.

#### 3.5.3. Playing an Active Role in the Birth

Finally, the mothers tried to play an active role in childbirth.

“*I had an important role in my childbirth. When I arrived at the ward, I interacted with a midwife and asked her some questions; I did whatever she asked me to help my childbirth” (Mo5, 28 years old, first birth).* Similarly*, “mothers are the cornerstone in normal childbirth and their participation in childbirth is important” (M8, 39 years old, ten years of work experiences).*

It is essential for mothers to play a role in their childbirths. Such a role may empower them in their family lives.

“It is important for me to help in the childbirth. As a mother, I should contribute to the childbirth, in addition to breeding baby” (Mo3, 24 years old, the second childbirth).

### 3.6. Consequences

#### 3.6.1. The Positive Experience of Childbirth

Action/interaction strategies taken by the participants resulted in different consequences. The positive experience of childbirth was the prominent consequence of actions/interactions in this study.

“I had a suitable relationship with a midwife, so I trusted her, and consequently, I had a good childbirth. I will select normal vaginal delivery if I become pregnant in future” (Mo12, 38 years old, second birth).

According to midwives and obstetricians, their performances can bring mothers a positive or negative experience of childbirth. They believe that mothers would choose a cesarean section in their future pregnancies if they had a negative experience of childbirth.

“A mother's experience of normal vaginal childbirth would depend on our performances and interactions. If we form a positive experience of childbirth in mothers' minds, surely they will select a normal vaginal delivery in future” (M9, 44 years old, with 29 years of work experiences).

## 4. Discussion

The present study revealed that mothers tried to trust healthcare providers. They applied three strategies of “mutual effective interaction,” “an attempt to access healthcare providers,” and “playing an active role in birth” to achieve this goal. In line with previous studies, our proposed visual model provides a framework for trust within the mother-midwife relationship in childbirth.

The mother-midwife relationship is based on mutual trust and assurance. Midwives should prepare the information needed for continuity of care and expand collaboration with mothers. Although such a partnership is essential and principle for mutual working, the role of the parties might not necessarily be equal [20]. Such a finding is also confirmed by Davis's model of shared decision making in midwifery [21]. Studies have shown that the reciprocal interaction between a midwife and a mother helps the mother identify her expectations and concerns during childbirth [22].

According to the participants' experiences, mothers should attempt to access healthcare providers. Stability of care means that mothers and midwives are competent to expand a secure interaction. This interaction is beyond the physical factors [23]. Some studies confirmed that the experience and availability of care providers [14, 15, 24], provision of demand-oriented childbirth care [25], and the women's right to select how they should be cared for [9] are the cornerstones of the quality care in maternal-infantile healthcare. These factors also provide the woman's safety, give her a sense of being supported, and induce an honorable childbirth experience [10, 25]. Then, the woman would satisfy with the care model [10].

The midwife's presence in the room with the mother does not necessarily mean that she recognizes maternal feelings and requests [7]. The concept of relationship-mediated being is defined as the combination of a midwife's being role and her individual, social, and empathic characteristics. It is the relationship-mediated being that allows the founding of a trusting relationship with the mother such that she feels listened to and understood [7, 9, 14].

Finally, a trustful relationship helps the mother have an active role in childbirth. Edmondson has reported that available facilities will affect the women's decision and plan and form a sense of being supported during normal vaginal childbirth [2]. Rudman has also reported that women sought a secure labor and birth process and concluded that adequate information helped them manage the situation and play an active role in their labors [26]. Regarding the continued process of care during pregnancy, birth, and the postnatal period, women know the importance of the midwife, identify her skills, and find a comfortable, friendly, and mutual interaction with her [27]. Previous studies have also reported that women select midwifery care based on their trusts in midwives' advocacies and sincerities. The caregiver's professionalism and compassion facilitate the women's trust in the midwife [22].

This study was conducted in Iran, so the findings might rarely be generalizable, but the results of this study could be generalized to societies with the same cultural backgrounds. Another limitation of this study is lack of interview with women's husbands and their family caregivers who were with mother during childbirth, and it is suggested that they be interviewed in future studies.

## 5. Conclusion

The present study showed that pregnant women tried to form a trustful relationship with midwives in childbirth by using strategies of “mutual effective interaction,” “an attempt to access healthcare provider,” and “playing an active role in birth”. The formation of trust was visualized in a model to help the healthcare providers in labor wards improve their performances. Such a model can facilitate a tool development to evaluate all midwifery, maternity, and infant care. Further multicenter research with a larger sample size may be necessary to prove these findings.

## Figures and Tables

**Table 1:** Characteristics of the participants.

Characteristics	Frequency (%)
*Women*		
*Age (years)*		
18	2 (16.67)	
18>	10 (83.33)	
*Parity*		
Primiparous	7 (58.33)	
Multiparous	5 (41.67)	
*Educational level*		
Elementary	2 (16.67)	
Secondary	4 (33.33)	
Postsecondary	3 (25.0)	
Postgraduate	3 (25.0)	
*Hospital of childbirth*		
Governmental	7 (58.33)	
Private	5 (41.67)	

*Midwives and obstetricians*		
*Educational level of midwives*		
BA degree	7 (63.63)	
MA degree	3 (27.27)	
PhD degree	1 (9.1)	
*Job experience* (*years*)	Midwives	Obstetricians
10>	5 (45.45)	3 (50)
10≤	6 (54.55)	3 (50)
*Type of hospital*	Midwives	Obstetricians
Governmental	8 (72.73)	4 (66.66)
Private	3 (27.27)	2 (33.33)

## Data Availability

The data are accessible by contacting the corresponding author.
